# Thylakoids reduce body fat and fat cell size by binding to dietary fat making it less available for absorption in high-fat fed mice

**DOI:** 10.1186/s12986-016-0160-4

**Published:** 2017-01-11

**Authors:** Karin G. Stenkula, Eva-Lena Stenblom, Caroline Montelius, Emil Egecioglu, Charlotte Erlanson-Albertsson

**Affiliations:** 1 Glucose Transport and Protein Trafficking, Department of Experimental Medical Science, BMC, Lund University, 221 84 Lund, Sweden; 2 Appetite Control, Department of Experimental Medical Science, BMC, Lund University, 221 84 Lund, Sweden

**Keywords:** PPARγ, PGC-1α, FAS, Adipose cell size, Fatty liver disease

## Abstract

**Background:**

Dietary thylakoids derived from spinach have beneficial effects on body fat accumulation and blood lipids as demonstrated in humans and rodents. Important mechanisms established include delayed fat digestion in the intestine, without causing steatorrhea, and increased fatty acid oxidation in intestinal cells. The objective of our study was to elucidate if increased fecal fat excretion is an important mechanism to normalize adipose tissue metabolism during high-fat feeding in mice supplemented with thylakoids.

**Methods:**

Mice were randomized to receive HFD or thylHFD for 14 days (*n* = 14 for the control group and 16 for the thylakoid group). The effect of thylakoids on body fat distribution, faecal and liver fat content, and adipose tissue metabolism was investigated following high-fat feeding.

**Results:**

Thylakoid supplementation for 14 days caused an increased faecal fat content without compensatory eating compared to control. As a result, thylakoid treated animals had reduced fat mass depots and reduced liver fat accumulation compared to control. The size distribution of adipocytes isolated from visceral adipose tissue was narrowed and the cell size decreased. Adipocytes isolated from thylakoid-treated mice displayed a significantly increased lipogenesis, and protein expression of peroxisome proliferator-activated receptor gamma (PPARγ), down-stream target FAS, as well as transcription factor coactivators PGC1-α and LPIN-1 were upregulated in adipose tissue from thylakoid-fed mice.

**Conclusions:**

Together, these data suggest that thylakoid supplementation reduces body fat and fat cell size by binding to dietary fat and increasing its fecal excretion, thus reducing dietary fat available for absorption.

## Background

Obesity is characterized by an excess fat mass due to chronic imbalance between energy intake and energy expenditure. Several comorbidities are associated with obesity, such as diabetes, cardiovascular disease and non-alcoholic fatty-liver disease (NAFLD). These comorbidities increase in prevalence at a high rate, underscoring an urgent need for strategies to prevent the emerging global epidemic [1].

An overload of energy in the form of dietary fat plays a significant role in promoting obesity compared to protein and carbohydrate [2]. Weight loss can therefore be achieved by reducing the consumption of fat or the absorption of fat. Lipase inhibitors are efficient in reducing body weight, however often with apparent gastrointestinal side effects such as steatorrhea [3].

Thylakoids are biological membranes, derived from green plants, that reversibly inhibit pancreatic lipase by binding to dietary fat droplets during fat digestion, thereby preventing access of the lipolytic enzymes present in the gastrointestinal tract [4]. In rodents long-term supplementation with thylakoids in high-fat diet (HFD) resulted in decreased body weight, decreased fat mass and decreased food intake [5]. It was not clear whether the reduction in food intake seen in these studies was in itself sufficient to explain the observed loss of fat mass [6]. There might also be a reduced fat absorption or an increased fatty acid oxidation. We however found no sign of steatorrhea in spite of a body weight loss, neither in rodent studies [7] nor in human studies [8, 9]. We also observed an increased expression of fat oxidative enzymes in the gut and an increased fat oxidation in rat fed high-fat diet with thylakoid supplementation [10], suggesting other mechanisms to explain the fat mass reduction. This could be fecal fat excretion and/or a recruitment of new adipocytes that have a more active metabolism. When storing energy, adipocytes initially grow in size, followed by differentiation and recruitment of new adipocytes [11, 12]. Special genes have been identified, the PPAR genes, which when activated increase the number of adipocytes so as to direct the energy to small adipocytes, which are less inflammatory [13].

In the present study we were interested in understanding the mechanism of action for a reduction of fat mass at the adipose cell level following thylakoid supplementation, by focusing on metabolism and cell size distribution of adipocytes [14]. We were also interested to find out if there was a fecal fat excretion that was not a full scale steatorrhea. To that end, mice were fed high-fat diet for two weeks and the adipocyte metabolism investigated as well as fecal fat excretion.

## Methods

### Animals

Ten to twelve week-old male C57Bl6 mice (Taconic, Denmark) weighing approximately 24–26 g upon arrival were used in the study. The animals were housed in rooms maintained on a 12/12-h light/dark cycle with lights on at 07:00 under stable conditions. The mice had *ad libitum* access to standard chow (R3, Lantmännen, Vadstena, Sweden) and tap water until the start of the experiments.

### Material

PPARγ, LPIN-1, FAS and PGC-1α antibodies were from Cell Signal Technology (Denvers, USA), β-actin antibody was from Sigma (Stockholm, Sweden).

### Thylakoids and diets

The chlorophyll-containing green-plant thylakoids used in the study were prepared from spinach leaves, as previously described [15], followed by drum drying. 100 g of thylakoids contain 23.5 g protein, 11.9 g fat, 41.7 g carbohydrate, 0.27 g sodium, 2.0 g chlorophyll, 27.9 mg lutein, 0.7 mg zeaxanthine, 4.8 mg beta-carotene, 0.021 mg vitamin A, 1.3 mg vitamin K, 6.0 mg vitamin E and 0.17 mg folic acid.

A high fat diet (HFD) and an isocaloric thylakoid-enriched HFD (thylHFD) were used in the experiments. The diets were designed to have the same macronutrient composition and the energy distribution of 46 E% fat, 18 E% protein and 36 E% carbohydrates. The diets were based on the D12451 high fat diet from Research Diets (Research Diets®, New Brunswick, NJ, USA). The thylHFD contained 33% w/w thylakoid extract (Appethyl®, Greenleaf Medical AB, Stockholm, Sweden), which is comparable to previous studies in rat that demonstrated effects on body weight and food intake [5–7].

### Experimental procedure

Upon arrival to the animal facility the mice were group housed two and two and were acclimatised to the facilities for a minimum of 7 days prior to the start of experiments. The mice were randomized based on body weight to receive HFD or thylHFD for 14 days (*n* = 14 for the control group and 16 for the thylakoid group). New food was administered twice a week and food consumption monitored. Body weight was measured day 0 (baseline), day 7 and at the end of the study on day 14. The mice were then euthanized and liver and body fat pads dissected and weighed and biopsies of adipose tissue taken for *in vitro* analysis of primary adipocytes.

### Dual-energy X-ray absorptiometry (DEXA)

To measure total body composition (total lean mass and fat mass), DEXA was performed on a subset of the animals (*n* = 6 and 9 for HFD and thylHFD fed groups respectively) using the Lunar PIXImus2™ DEXA scan (GE Healthcare, WI, USA).

### Faecal fat content

Faecal samples were collected from the mice (*n* = 14 (control) and 16 (thylakoid)) during the last 8 days of the two-week period and dried for later analysis. The faecal samples were analyzed for fat content using acid hydrolysis (Eurofins Food & Feed testing laboratory, Linköping, Sweden).

### Liver triacylglycerol (TG) assay

Snap-frozen liver tissues were homogenized 1:4 in 50 mM Tris–HCl pH 7.5, 1 mM EGTA, 1 mM EDTA, 0.27 M sucrose, 5% NP-40, 1 mM dithiothreitol (DTT) and complete protease and phosphatase inhibitor. Samples were submitted to a cycle of heating (85 °C for 5 min, room temperature for 10 min, and 85 °C for 5 min) and centrifugation at 16000 xg for 2 min. The supernatant was assayed using Infinity TG Liquide Stable Reagent (Thermo Scientific, USA).

### Cell size distribution

Adipose cell-size distribution was measured using a Beckman-Coulter counter after osmium fixation as described previously [14, 16]. In brief, 10–20 mg of epididymal adipose tissue sample (*n* = 6 samples/group) were fixed in 1% osmium for 48 h, followed by washing with NaCl. Adipose cell size was determined by a Beckman Coulter Multisizer III with a 400 μm aperture. The instrument was set to count 6,000 particles, and the fixed-cell suspension was diluted so that coincident counting was <10%. After collection of pulse sizes, the data were expressed as particle diameters and displayed as histograms of counts against diameter using linear bins and a linear scale for the x-axis.

### Adipose cell isolation and lipogenesis assay

To measure lipogenesis, primary mouse adipocytes were isolated from epididymal and mesenteric fat tissue as described previously [17]. Lipogenesis was measured according to a previous method [18]. In brief, adipocytes were re-suspended in Krebs-Ringer (KRH) medium containing 25 mM Hepes pH 7.4, 200 nM adenosine, 0.55 mM glucose and 3.5% BSA (w/v). In triplicates, 700 μl of the 2%(v/v) adipocyte cell suspension was added to each tube and incubated for 1-h at 37 °C with 14 μl of 22 μCi/ml tritiated glucose (D-[6-3H]-glucose, Perkin Elmer, Cambridge, UK) either with or without 28 nM insulin. After the incubation, the assay was stopped by adding 3.5 ml of 2,5-diphenyloxazole (Sigma-Aldrich, Stockholm, Sweden) and 1,4-bis (5-phenloxazol-2-yl) benzene (Sigma) toluene-based scintillation liquid (Sigma). A zero sample was also included in the experiment to measure how much glucose ends up in the lipid phase during extraction without having been used for lipid synthesis. This was done by adding 700 μl of the 2% adipocyte cell suspension to scintillation tubes containing 14 μl of 22 μCi/ml tritiated glucose which was then stopped immediately by adding 3.5 ml PPO-POPOP toluene-based scintillation liquid.

### Western blot

Intact adipose tissue was homogenized with a polytron (Omni International TH) in lysis buffer containing 50 mM Tris/HCl pH 7.5, 1 mM EGTA, 1 mM EDTA, 1 mM sodium orthovanadate, 10 mM sodium-β-glycerophosphate, 50 mM sodium fluoride, 5 mM sodium pyrophosphate, 0.27 M sucrose, 1% NP-40, 1 mM dithiothreitol (DTT) and complete protease inhibitor cocktail (one tablet/50 ml) (lysis buffer). Lysates were centrifuged for 15 min at 10000 × g and protein concentrations were determined by Bradford [19]. Adipose tissue lysates were heated at 95 °C for 2 min in SDS sample buffer, and subjected to polyacrylamide gel electrophoresis on pre-cast BioRad gradient gels and electro transfer to nitrocellulose membrane. An amount of ten μg protein was loaded per sample according to Bradford protein quantification. Membranes were blocked for 30 min in 50 mM Tris/HCl pH 7.6, 137 mM NaCl and 0.1% (w/v) Tween-20 (TBS-T) containing 10% (w/v) milk powder. The membranes were then probed with indicated antibodies in TBS-T containing 5% (w/v) milk or 5% (w/v) BSA, for 16 h at 4 °C. Detection was performed using horseradish peroxidase conjugated secondary antibodies and the chemiluminescence reagent. The signal was visualized using a BioRad Image camera and band intensities quantified using BioRad Imaging software (Biorad, CA, USA).

### Statistical analyses

Statistical analyses were done using Prism GraphPad version 6 (GraphPad Software Inc. La Jolla, CA, USA). Not assuming Gaussian distribution, Kruskal-Wallis statistic and unpaired Mann–Whitney tests was used to determine statistical differences. All data are presented as median and interquartile range if not otherwise mentioned. *P*-values < 0.05 were considered to be statistically different.

## Results

### Body weight gain and food intake

**Fig.1:**
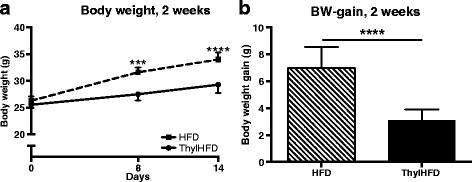
**a**-**b** Body weight development **a** and body weight gain **b** during the two-week study. Bars represent median and interquartile range. Statistical difference of *p* < 0.05 was considered significant (*** = *p* < 0.001, **** = *p* < 0.0001)

To examine the effect of thylakoids on body weight and fat mass, mice were fed HFD or thylHFD for two weeks. As expected, mice in both groups displayed significant (*p* < 0.0001) body weight gain, which however was suppressed in mice fed thylHFD compared to HFD mice at the end of the study (*p* < 0.0001, Fig. .1a and b). A difference in body weight was noted already after one week (*p* < 0.001) and continued during the second week of feeding (Fig. .1a and b). Mice fed the thylHFD showed reduced caloric intake compared to HFD-fed mice (*p* < 0.05, Table I).

### Body fat

**Fig. 2:**
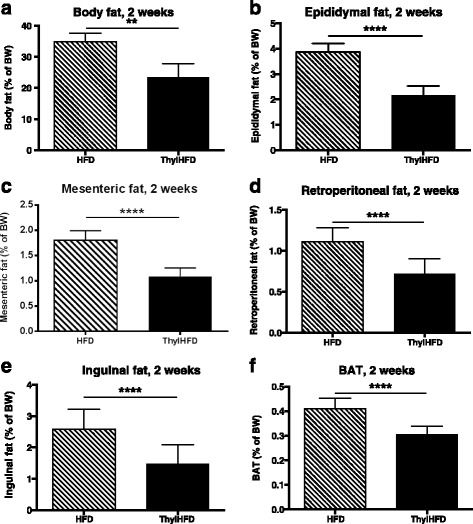
**a**-**f** Measurements of body fat accumulation after two weeks in mice fed HFD vs. ThylHFD. Total body fat (%of BW measured by DEXA, **a**), Epididymal fat **b**, Mesenteric fat **c**, Retroperitoneal fat **d**, Inguinal fat **e** and Brown adipose tissue **f**. Bars represent median and interquartile range. Statistical difference of *p* < 0.05 was considered significant (**p < 0.01, ***p < 0.001, ****p < 0.0001)

After two weeks on HFD, DEXA-analysis revealed a reduced fat mass by 33% in the thylHFD-fed mice compared to mice fed HFD (*p* < 0.01) (Fig. 2a). There was no difference in lean mass (data not shown). Analysis of individual fat depots demonstrated a reduction in epididymal fat (*p* < 0.0001, Fig. 2b), mesenteric fat (*p* < 0.0001, Fig. 2c), retroperitoneal fat (*p* < 0.0001, Fig. 2d), inguinal fat (*p* < 0.0001, Fig. 2e) and BAT (*p* < 0.0001, Fig. 2f) in thylHFD-fed mice compared to HFD.

### Faeces analysis

Analysis of fat in the faeces demonstrated that the mice receiving thylakoids had significantly higher lipid elimination through the faeces than high-fat fed control animals (Table 1), the fat content being 23 mg per day in the thylakoids-fed animals and 10 mg per day in the controls.

**Table 1:** Impact of high-fat diet with or without thylakoids on food intake and lipid excretion

	Control HFD diet	Thylakoid HFD
Food intake (g/day)	3.60 (3.52–3.84)	3.72 (3.44–3.87)
Caloric intake (kcal/day)	16.76 (16.41–17.91)	16.05 (14.88–16.71)^a^
Total lipid content in feces (mg/day)	9.60 (8.69–10.42)	22.73 (19.25–25.37)^b^

Values are shown as median and interquartile range (*n* = 14 control and 16 thylakoid). ^a^ = *p* < 0.05, ^b^ = *p* < 0.001, Mann–Whitney test

### Liver fat content

**Fig. 3:**
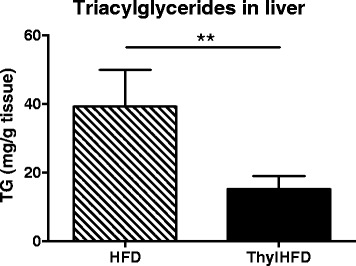
Liver fat accumulation in high-fat fed control and high-fat fed thylakoid animals (mg triacylglycerides (TG)/mg tissue). Statistical difference of p < 0.05 was considered significant (**p < 0.01)

Livers from mice fed thylHFD had decreased fat (TG) accumulation compared to the HFD-fed mice (*p <* 0.001, Fig. 3). There was no difference in liver weight between thylHFD-fed mice and HFD (*p* = 0.185, data not shown).

### Lipid metabolism in adipocytes

**Fig. 4:**
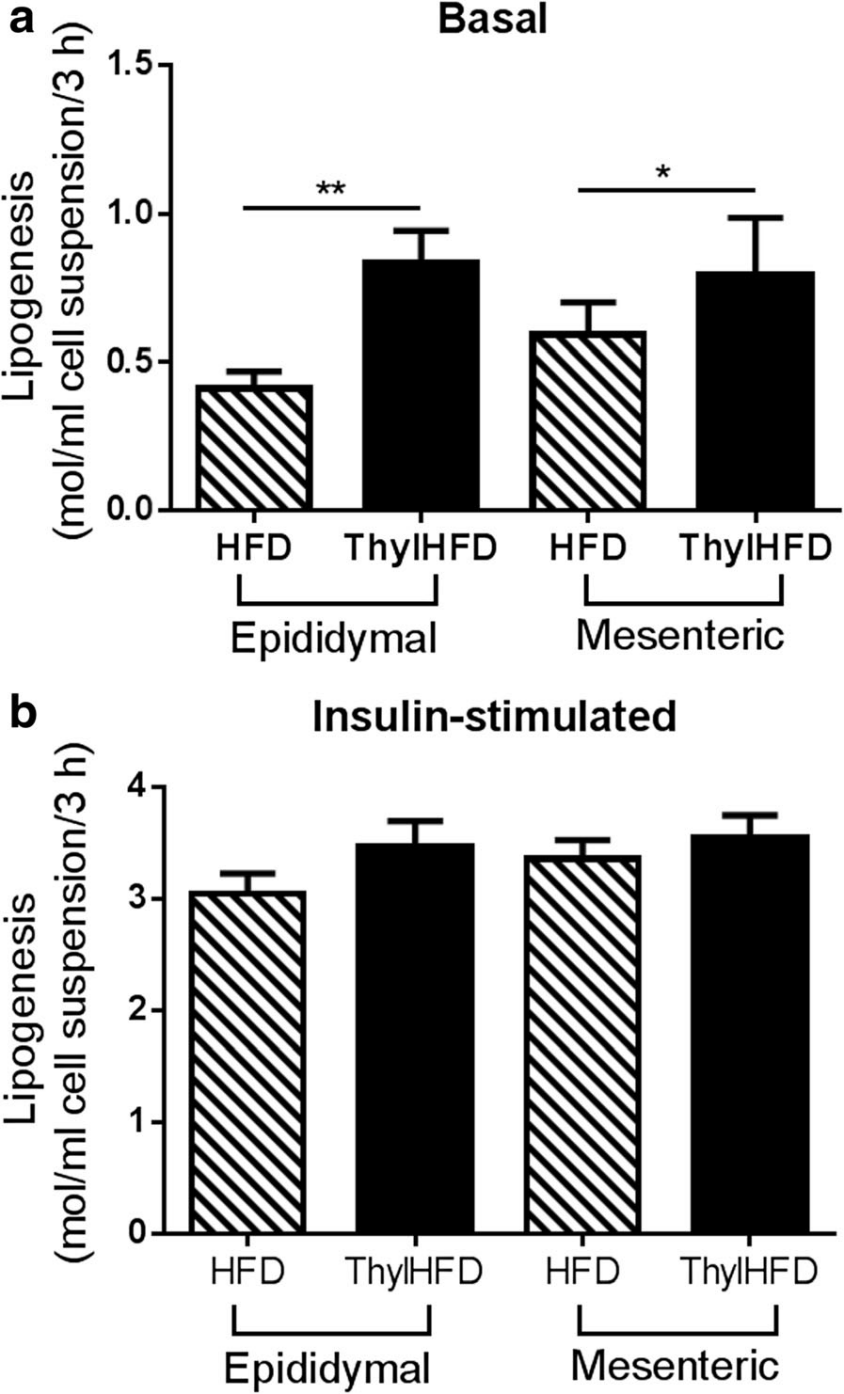
**a**-**b** Lipogenesis in the basal (non-stimulated) **a** and insulin-stimulated (28 nM) **b** states in epididymal and mesenteric adipocytes from mice fed HFD vs ThylHFD for two weeks. Bars represent median and interquartile range. Statistical difference of *p* < 0.05 was considered significant (*p < 0.05, **p < 0.01)

Lipogenesis was measured using the incorporation of tracer glucose into the triglycerides. In the non-stimulated state, lipogenesis was increased both in cell-isolates from mescenteric (*p* < 0.05) and epididymal fat (*p* < 0.01) from the thylHFD mice compared with HFD-fed mice (Fig. 4a). Insulin-stimulated lipogenesis was similar in cells isolated from thylHFD-fed and HFD-fed mice comparing both fat depots (Fig. 4b).

### Cell size of adipocytes

**Fig. 5:**
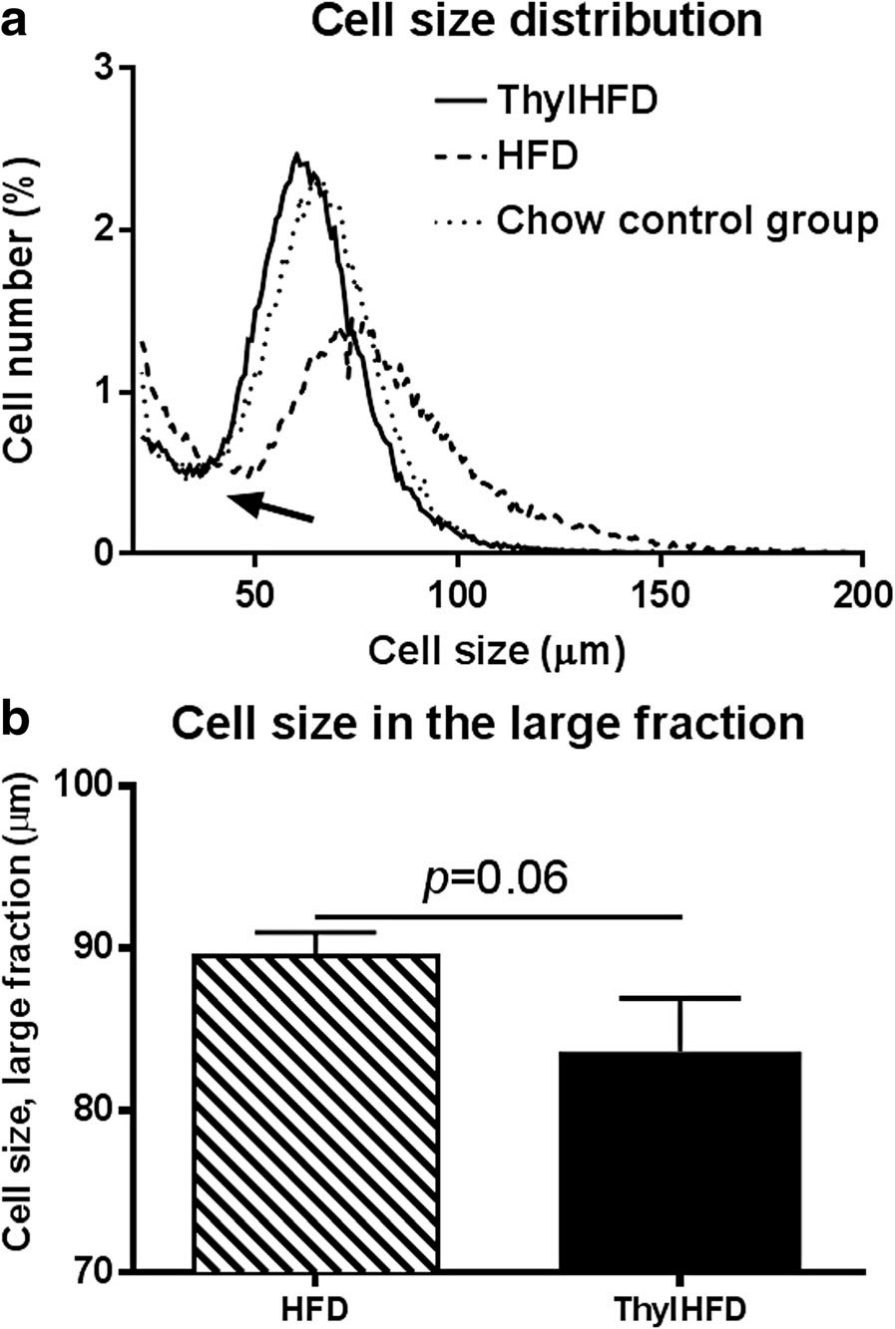
**a**-**b** Adipocyte size distribution **a** in large versus small cell fraction (defined as above and below the nadir point, marked with arrow), dotted line represents previous reported cell size distribution of chow-fed mice (chow control group) [12], here inserted for comparison. The mean cell size of the cells in the large fraction **b** from mice fed HFD vs ThylHFD for two weeks. Bars represent median and interquartile range. Statistical difference of *p* < 0.05 was considered significant (**p < 0.01)

In both groups, there was a bimodal cell distribution, with a fraction of so-called small cells defined by the nadir [16], the lowest point between the two distributions (indicated with an arrow in Fig. 5a), and a cell population of larger cells, so-called large cells, to the right of the nadir. In the HFD group, the large cells were varying in size between 50–150 μm diameter, with a mean cell size of 90 μm (Fig. 5b). In the thylakoid treated animals, the cell size distribution was markedly shifted to the left and more narrowed, with large cells varying between 30–100 μm, with a decreased mean cell size (~83 μm) (Fig. 5b). Overall, the cell size distribution of thylHFD fed mice was comparable with the distribution curve from chow-fed mice, reported previously [12] (Fig. 5a).

### PPARγ, PGC-1α, LPIN-1 and FAS expression in epididymal fat tissue

**Fig. 6:**
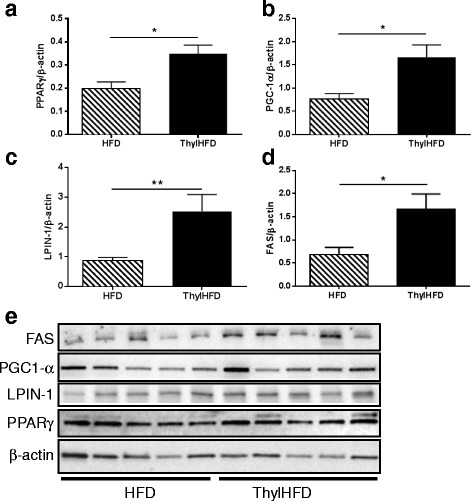
**a**-**e** Quantification of Western blot analysis of PPARγ **a**, PGC1-α **b**, LPIN-1 (Fig. **c**), and FAS **d** in WAT (*n* = 5 animals/group). β-actin was used for normalization. Western blot images shown in **e**. Bars represent median and interquartile range. Statistical difference of *p* < 0.05 was considered significant (*p < 0.05, **p < 0.01)

Quantification of Western blot analysis demonstrated that thylakoid supplementation increased the protein expression of transcription factor PPARγ (Fig. 6a), transcription factor coregulatory elements PGC-1α (Fig. 6b), LPIN-1 (Fig. 6c), and down-stream target FAS (Fig. 6d). Western blotting data used for quantification (Fig. 6e). Protein expression was normalized to expression of β-actin.

## Discussion

In this work we have demonstrated that green-plant thylakoids protect against body fat accumulation due to high-fat feeding in mice. As a novel finding, an increased faecal fat excretion was observed in the thylakoid-fed animals, amounting to 23 mg per day in comparison to 10 mg in the control group. This is the first time green-plant thylakoids were found to enhance fecal excretion of fat and energy, which thus substantiates the well-known finding of thylakoids as potent inhibitors of pancreatic lipase activity by binding to dietary lipids [5]. There was thus a substantial amount of fat in the faeces, without reaching the levels of steatorrhea, defined as a minimal fecal fat excretion of 35 mg per day in mouse [20]. The increased fecal fat excretion may explain the observed body weight loss and body fat loss in the thylakoid-fed mice in these studies, but also in previous studies where adiposity in mice measured with DEXA were found to decrease significantly [6].

Other lipase inhibitors like orlistat used for treatment of obesity increases fecal fat, in humans to 16 g per day, a level which is three-fold higher than allowed levels. Such a high excretion of fat may lead to gastrointestinal adverse events that were also found by the majority in the treatment group [21]. The excretion of fecal fat may however be an important mechanism to induce body weight and body fat loss, as observed by Orlistat treatment [21]. Chitosan with a potential to block fat absorption fails to induce body weight loss [22] and does not either cause any increased fecal fat content [23].

Fecal fat excretion may lead to overeating, as observed in colipase-deficient mice having a massive steatorrhea [24]. However, we found no compensatory overeating (Table 1), which suggests other functional properties of thylakoid membranes to stabilize body weight. Such properties may be an increased release of satiety hormones like CCK and GLP-1, as observed in rodents [6] and in humans [25, 26]. An increased release of satiety hormones suggests a delayed absorption of nutrients from the intestine during digestion through thylakoid membranes. In previous publications, we have found no evidence of increased faecal fat excretion by the addition of thylakoids despite body weight and fat mass loss, neither in man [9], nor in rat [7]. The thylakoid enriched high-fat diet used in mice was the same as previously used for rat, suggesting a species difference. In rat, the absence of fat in faeces could be due to a slower passage of food through the intestine [9], giving the possibility for an increased intestinal fatty acid oxidation [10] and/or a more complete fat absorption.

A reduced fat absorption due to thylakoid supplement as found here, decreased fat mass and also changed the adipose cell size distribution (ranging between 30–100 μm in the thylakoid-high-fat fed animals, compared to 50–150 μm in the control high-fat fed animals (Fig. 5). Indeed, the size of the adipocytes resembled those measured in mice fed a low-fat diet (Fig. 5). In parallel, we observed in the thylakoid fed animals observed an upregulation of PPARγ, its down-stream target FAS, and of PGC-1γ and LPIN-1, nuclear transcriptional coactivators of PPARs, which stimulate the differentiation of new adipocyte precursors and promote lipogenesis [27]. In our study we could not determine the activity of the nuclear receptor PPARγ, to support that an increased protein expression also meant an increased activity. We hypothesize that thylakoid supplementation could stimulate lipid storage in small cells by recruitment of precursors (hyperplasia), rather than expanding already large cells (hypertrophia). This is important since enlarged cells are associated with inflammation and impaired metabolic homeostasis.

An increased basal lipid storage capacity was observed in both mesenteric and epididymal adipocytes isolated from mice fed thylakoids (Fig 4.). Thus, increased PPARγ, PGC-1α and LPIN-1 expression together with the distribution profile, and an increased expression of the enzyme FAS which is central for lipid metabolism, support the hypothesis of an increased recruitment of metabolically active adipocyte precursors that could explain the reduced fat mass even with increased lipogenesis. Investigations have identified several natural agonists for PPARγ, bioactive compounds being plant antioxidants such as luteolin, quercitin, catechin and resveratrol [28]. Thylakoids contain antioxidants in large amounts, like lutein, karotenoids and zeaxantin. Therefore, we also hypothesize that these antioxidants may be responsible for an interaction with the PPARγ system.

In this study, ThylHFD-fed mice also accumulated less fat in the liver compared to HFD-fed mice. Earlier studies have demonstrated reduced levels of triacylglycerol in the blood by thylakoids [4, 6], but this is the first time that we observe a reduction of fat accumulation in the liver. This finding may be of clinical importance, since NAFLD is a metabolic disorder and a multi-system disease, strongly associated with obesity, insulin resistance and type-2 diabetes [29], the incidence of NAFLD increasing at an alarming rate [30]. Due to its effects on hepatic oxidative capacity, circulating TG and free fatty acid levels, LPIN-1 has been suggested as a potential therapeutic target for obesity-related dyslipidaemia and NAFLD [30]. Possibly, treatment with thylakoids increases LPIN-1 expression in liver similar to adipocytes that may reduce fat accumulation.

## Conclusions

In conclusion we have demonstrated that thylakoids when given to mice fed a high-fat diet reduce food intake while increasing fecal fat excretion without causing steatorrhea. This led to reduced body weight, fat mass and liver fat accumulation. Adipocytes were found to be reduced in size and possibly these correspond to a cell population that is more insulin sensitive than larger adipocytes. Further studies are needed to find out if there is a change in fat cell size and increased insulin sensitivity also in humans.

## Abbreviations

NAFLD: Non-alcoholic fatty-liver disease
HFD: High-fat diet
PPARγ: Peroxisome proliferator-activated receptor gamma
DEXA: Dual-energy X-ray absorptiometry
TG: Triacylglycerol
